# Supplementary material for: In Vitro Synergistic Inhibitory Effects of Plant Extract Combinations on Bacterial Growth of Methicillin-Resistant Staphylococcus aureus
Source: Pharmaceuticals (Basel). 2023 Oct 20;16(10):1491. doi: 10.3390/ph16101491 (PMC10610001; doi:10.3390/ph16101491)
Supplement: Supplementary file 1 [file pharmaceuticals-16-01491-s001.zip › pharmaceuticals-2657072-supplementary.pdf]

Supplementary Materials

In Vitro Synergistic Inhibitory Effects of Plant Extract Combinations on Bacterial Growth of Methicillin-Resistant *Staphylococcus aureus*

Jea-Young Jeong, In-Geun Jung, Seung-Hoon Yum, You-Jin Hwang

Table S1. UPLC mobile phase gradient conditions

Time (min)	Flow rate (mL/min)	Phase A (%)[a]	Phase B (%)[b]
0.0	0.40	92	8
1.0	0.40	92	8
16.0	0.40	30	70
17.0	0.40	0	100
19.0	0.40	0	100
19.3	0.40	92	8
22.0	0.40	92	8

[a] Phase A: Distilled water with 0.1% formic acid.

[b] Phase B: Acetonitrile with 0.1% formic acid.

Table S2. Mass spectrometer operating conditions

Parameters	Values
Desolvation gas	N_2
Desolvation flow rate	800 L/h
Desolvation temperature	350 °C
Source temperature	110 °C
Capillary voltage	300 V
Cone voltage	40 V
Scan mode*	ESI^- / ESI^+
m/z range	100-1500 Da

* ESI^-: Negative electrospray ionization / ESI^+: Positive electrospray ionization.

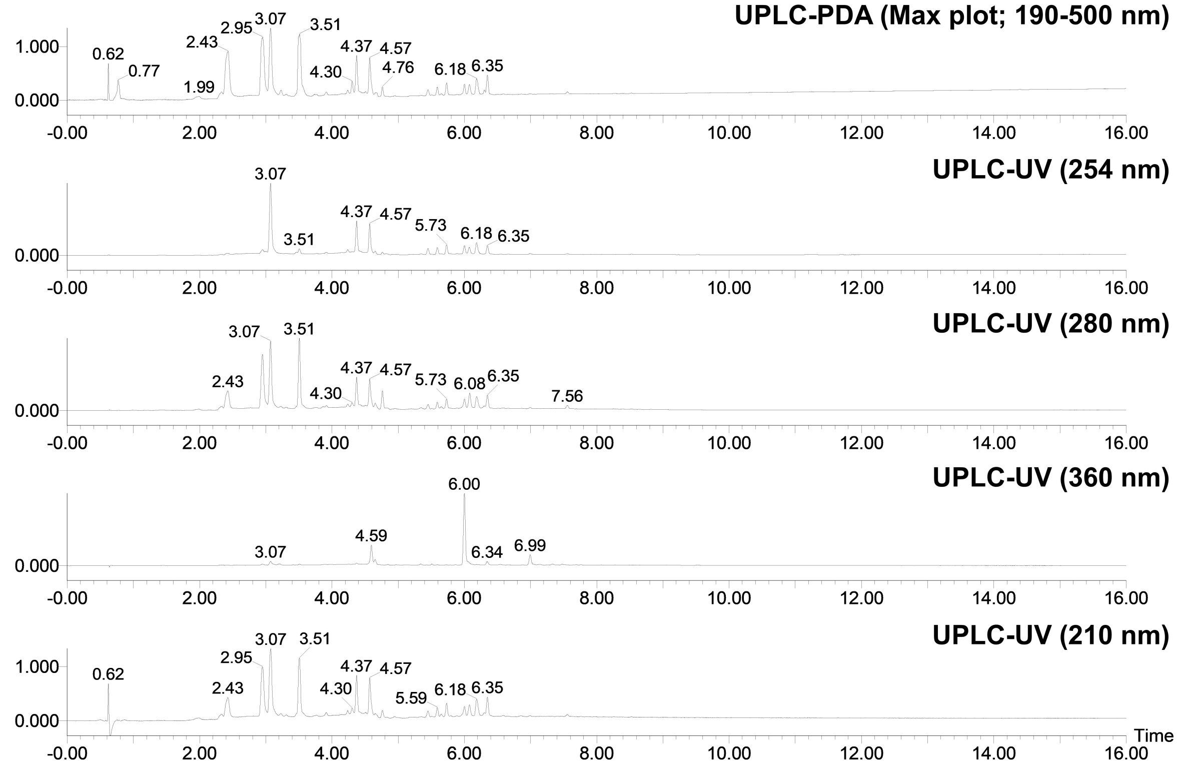

Supplementary Figure S1. UPLC chromatograms of 70% ethanol extracts (3 mg/mL) from *Caesalpinia sappan* L. The analysis conditions were set as follows: Column, ACQUITY UPLC BEH C18 1.7 µm column (2.1 x 100 mm); column temperature, 35 °C; flow rate, 0.4 mL/min; sample injection volume, 1 µL; detection wavelength , max plot (190-500 nm), 210 nm, 254 nm, 280 nm, and 360 nm. The mobile phase gradient conditions were set as described in Table S1.

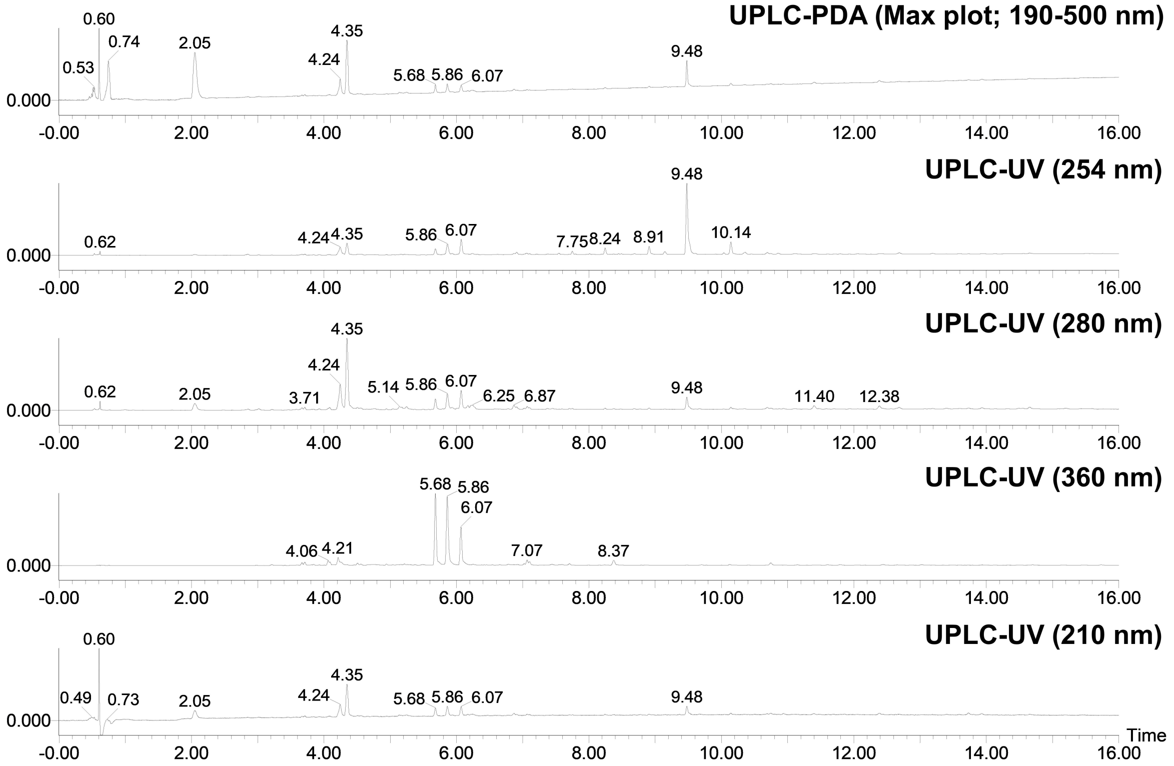

Supplementary Figure S2. UPLC chromatograms of 70% ethanol extracts (3 mg/mL) from *Glycyrrhiza uralensis* Fisch. The analysis conditions were set as follows: Column, ACQUITY UPLC BEH C18 1.7 μm column (2.1 x 100 mm); column temperature , 35 °C; flow rate, 0.4 mL/min; sample injection volume, 1 μL; detection wavelength, max plot (190-500 nm), 210 nm , 254 nm , 280 nm , and 360 nm . The mobile phase gradient conditions were set as described in Table S1.

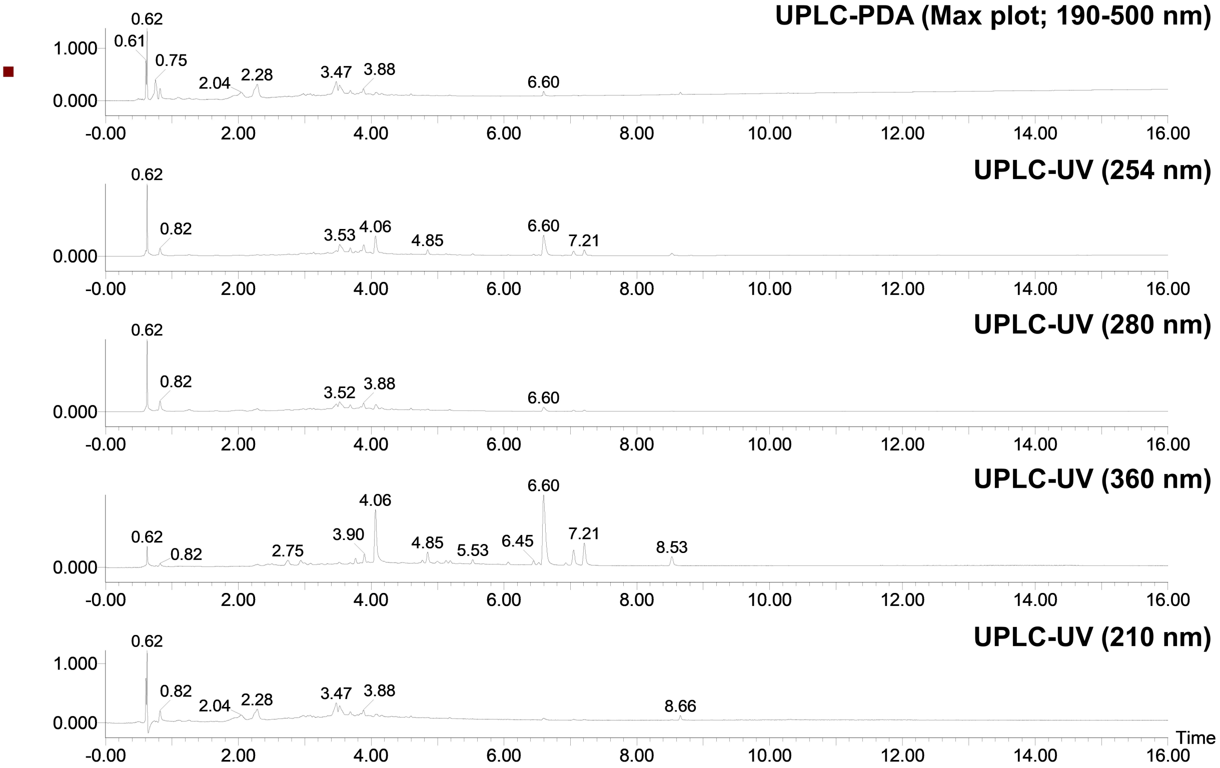

Supplementary Figure S3. UPLC chromatograms of 70% ethanol extracts (3 mg/mL) from *Sanguisorba officinalis* L. The analysis conditions were set as follows: Column, ACQUITY UPLC BEH C18 1.7 μm column (2.1 x 100 mm); column temperature, 35 °C; flow rate, 0.4 mL/min; sample injection volume, 1 μL; detection wavelength, max plot (190-500 nm), 210 nm , 254 nm , 280 nm , and 360 nm . The mobile phase gradient conditions were set as described in Table S1.

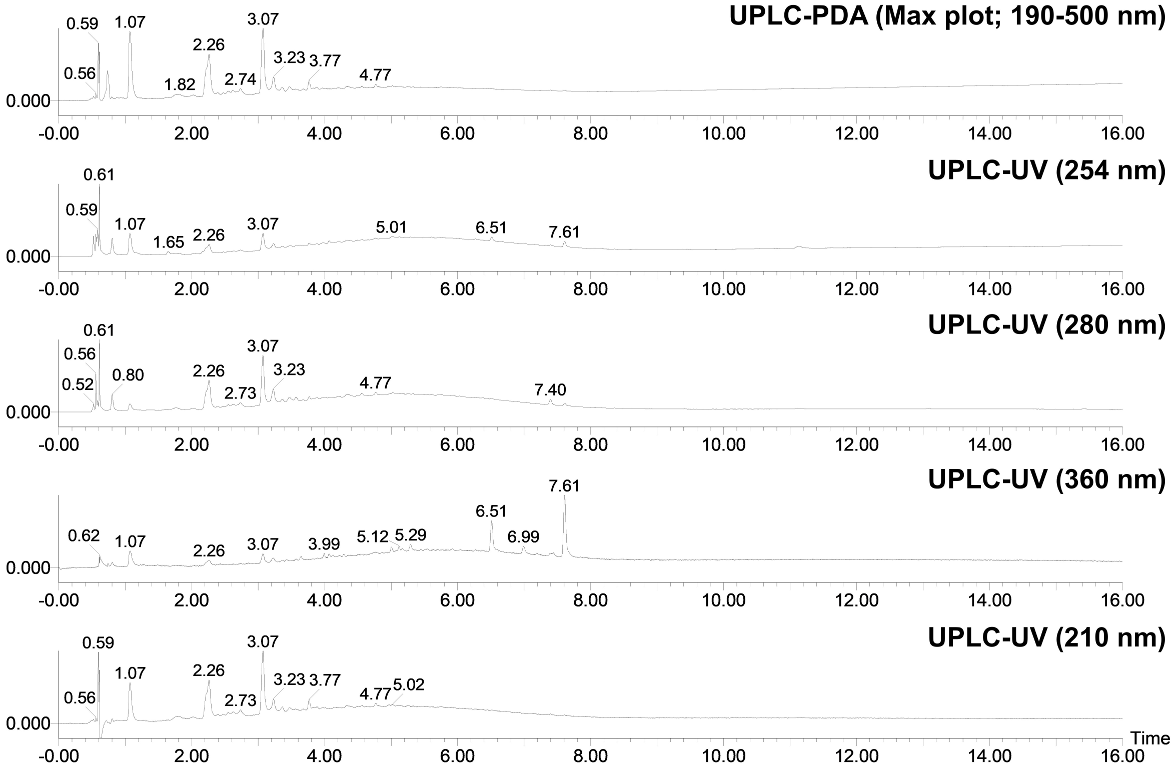

Supplementary Figure S4. UPLC chromatograms of 70% ethanol extracts (3 mg/mL) from *Uncaria gambir* Roxb. The analysis conditions were set as follows: Column, ACQUITY UPLC BEH C18 1.7 μm column (2.1 x 100 mm); column temperature, 35 °C; flow rate, 0.4 mL/min; sample injection volume, 1 μL; detection wavelength, max plot (190-500 nm), 210 nm , 254 nm , 280 nm , and 360 nm . The mobile phase gradient conditions were set as described in Table S1.

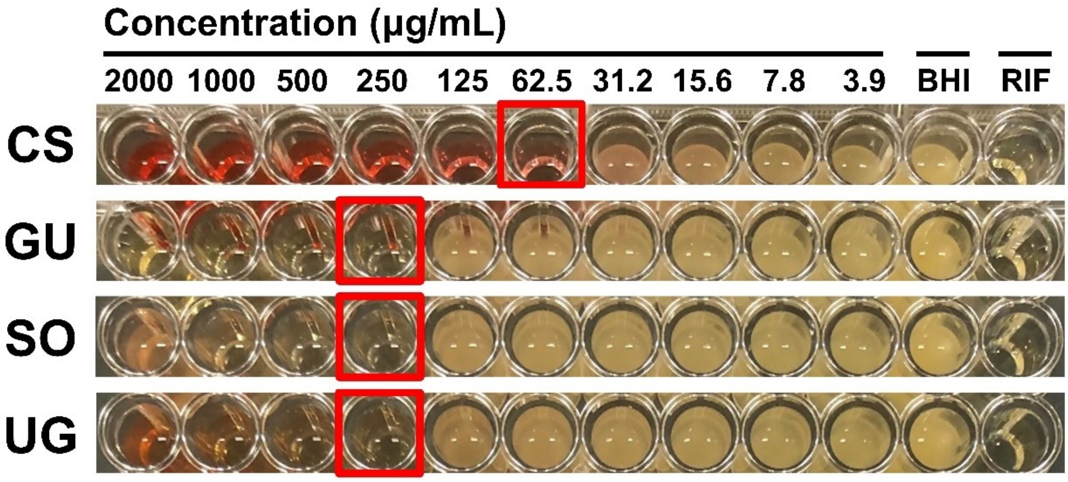

Supplementary Figure S5. Determination of minimum inhibitory concentration (MIC) of selected medicinal plants using broth microdilution method. CS: *Caesalpinia sappan* L.; GU: *Glycyrrhiza uralensis* Fisch.; SO: *Sanguisorba officinalis* L.; UG: *Uncaria gambir* Roxb. Red squares indicate the MICs of ethanol extracts. Brain heart infusion (BHI) broth was served as a negative control, while rifampicin (RIF; 0.08 μg/mL) was a positive control.

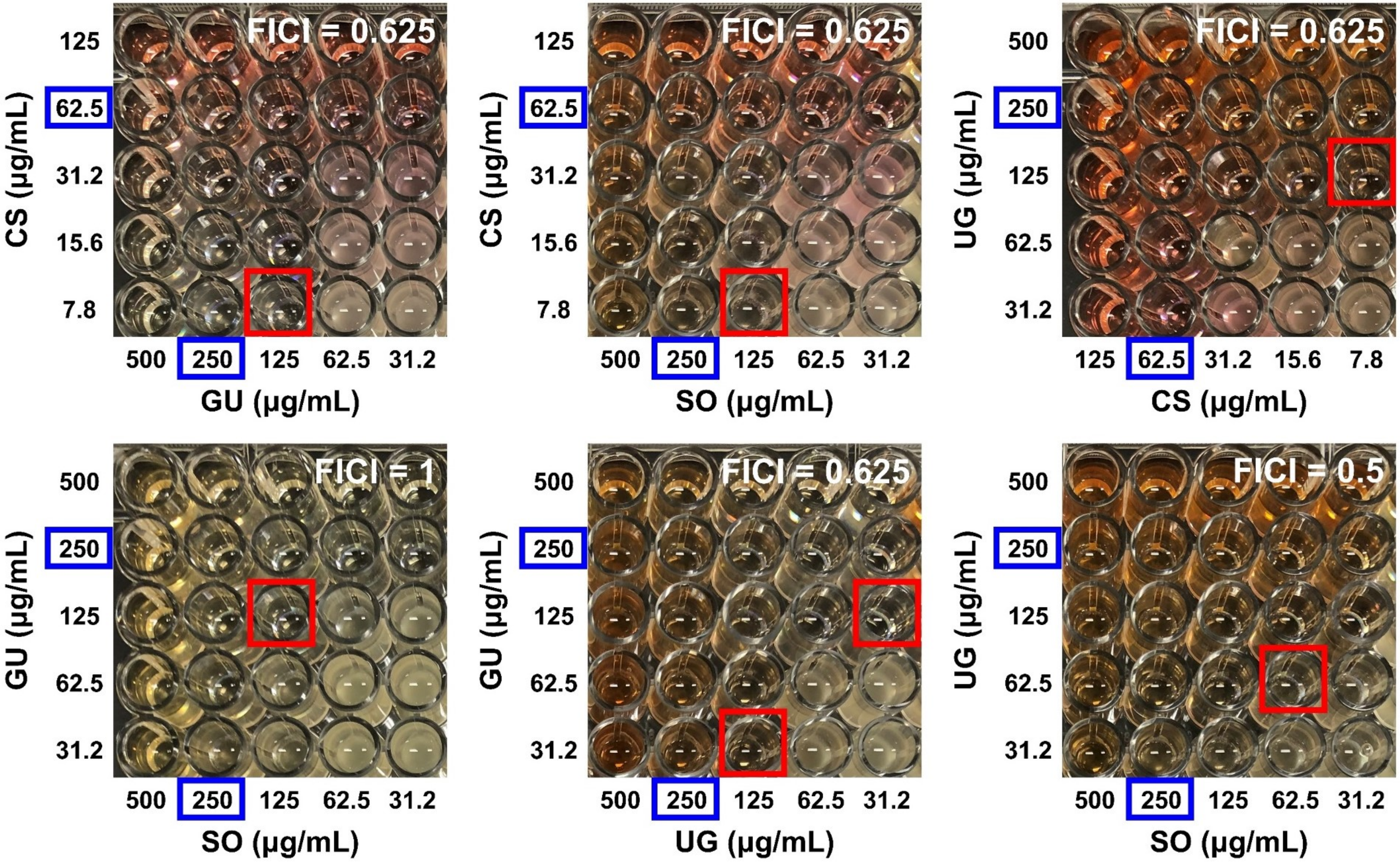

Supplementary Figure S6. Evaluation of synergistic inhibitory effects of plant extract combinations by checkerboard synergy assays. CS: *Caesalpinia sappan* L.; GU: *Glycyrrhiza uralensis* Fisch.; SO: *Sanguisorba officinalis* L.; UG: *Uncaria gambir* Roxb. Red squares indicate the MICs of plant extract combinations. Blue squares indicate the MICs of individual extracts.
